# Urushiol-Based Coating with High Surface Hydrophilicity for Easy-Cleaning of Oil Pollutants

**DOI:** 10.3390/polym16233392

**Published:** 2024-11-30

**Authors:** Yuansong Ye, Huiping Shi, Yuchi Zhang, Jianrong Xia, Bing-Chiuan Shiu, Run Fang

**Affiliations:** 1 College of Materials and Chemical Engineering, Minjiang University, Fuzhou 350108, China; yeyuansong@mju.edu.cn (Y.Y.); 3223103206@stu.mju.edu.cn (H.S.); zyc@mju.edu.cn (Y.Z.); jrxia@mju.edu.cn (J.X.); 2 Fujian Engineering and Research Center of New Chinese Lacquer Materials, Minjiang University, Fuzhou 350108, China

**Keywords:** coating, urushiol, polyethylene glycol monooleate, high surface hydrophilicity, easy-cleaning property

## Abstract

Urushiol is recognized as a sustainable coating material with superior properties; however, it faces significant challenges in applications such as petrochemicals and marine engineering due to surface oil contamination. This study aimed to enhance the cleanability of urushiol-based coatings through hydrophilic modification. Polyethylene glycol monooleate (PEGMO) was identified as an appropriate hydrophilic macromonomer and utilized as a modifier to develop a novel urushiol-based coating, termed P(U-PEGMO), via thermal curing. The results indicated that copolymerization occurred between urushiol and PEGMO during the curing process, forming a stable urushiol copolymer with favorable compatibility. The incorporation of PEGMO greatly improved the surface hydrophilicity of the coatings, as evidenced by a reduction in the water contact angle to below 30° when the modifier content reached 30% or higher, demonstrating a high degree of surface hydrophilicity. This enhanced property imparted the modified coating with underwater superoleophobicity and reduced oil adhesion, thereby facilitating the removal of oil. The cleaning performance was evaluated using a simple water rinsing method, after which, less than 2.5 wt% of oil residues remained on the surface of the modified coating. The high hydrophilicity is considered responsible for the coating’s easy-cleaning capability. In addition, the modified coatings exhibited improved flexibility and impact resistance, albeit with a slight decrease in hardness.

## 1. Introduction

Oriental lacquer, a high-quality natural coating derived from the phloem of lacquer trees, constitutes a valuable resource provided by nature [1,2,3]. The main film-forming component of this lacquer is urushiol, a catechol derivative characterized by a long side chain, typically composed of C_15_ or C_17_ straight-chain aliphatic hydrocarbon containing 0–3 C=C bonds. Due to its multiple active functional groups, urushiol can undergo enzymatic polymerization at room temperature catalyzed by laccase, or thermal curing at high temperatures without laccase, forming complex cross-linked polymer coatings [2,4,5]. These urushiol-based coatings exhibit exceptional physicochemical properties and environmental compatibility, rendering them increasingly valuable across various industries, including petrochemicals, offshore oil and gas development, as well as printing and dyeing [3,6,7,8,9]. Common applications include the coating of chemical pipelines, storage tanks, offshore drilling equipment, and marine vessels. However, urushiol-based coatings are prone to surface contamination by oil-based organic substances present in the surrounding environment, such as crude oil, fuel oil, lubricants, and other chemicals. This oil contamination can deteriorate the coatings’ weather resistance, reduce adhesion, and degrade their appearance, necessitating regular maintenance. Traditional cleaning methods such as steam, detergents, and chemical solvents [10], while effective in removing oil pollutants, often involve complex procedures and risk damaging the coating or causing environmental harm. Consequently, the development of innovative strategies for the removal of oil contamination from urushiol-based coatings is of considerable significance.

The surface wettability of solid materials is a critical determinant of their cleaning performance. Hydrophilic surfaces, characterized by their strong affinity with water and low contact angles, demonstrate excellent easy-cleaning properties that can be achieved through simple, effective, and environmentally friendly cleaning methods [11,12]. The interaction between water and a hydrophilic surface allows for the formation of a dense hydration layer that repels oil and facilitates the replacement of the oil layer with water, enabling easy oil removal through rinsing [11]. The stronger the surface hydrophilicity, such as highly hydrophilic or superhydrophilic surfaces, the better the cleaning performance against oil pollutants [13,14,15]. In contrast, urushiol-based coatings typically exhibit weak surface hydrophilicity due to the presence of nonpolar groups (e.g., benzene rings and aliphatic hydrocarbons) in their chemical structure [16]. Therefore, enhancing the hydrophilicity of urushiol-based coatings represents a promising strategy to improve their cleaning properties and effectively address surface contamination.

Among the various methods for the hydrophilic modification of polymers, chemical copolymerization is particularly effective due to its ability to impart enduring hydrophilic properties and its broad range of applicability [17]. It can overcome issues encountered in physical modification techniques, such as poor compatibility between modifiers and substrates and the agglomeration of modifiers, which can reduce hydrophilization and durability [17,18]. For urushiol, hydrophilic copolymerization could theoretically occur at room temperature with laccase or at high temperatures through thermal curing, corresponding to its two primary curing methods. However, room-temperature copolymerization is less feasible for urushiol due to its requirement for high humidity and extended curing times [1], which hinder the participation of modifiers in the curing reaction. Thus, high-temperature copolymerization emerges as the preferred approach. Urushiol’s thermal curing involves complex mechanisms, including the condensation of phenolic hydroxyl groups and oxidative/addition polymerization of the C=C bonds in the side chains [1,4]. This suggests that selecting hydrophilic vinyl monomers likely facilitates copolymerization with urushiol’s unsaturated side chain during curing. Currently used vinyl monomers for hydrophilic modification include acrylic acid (AA), acrylamide (AM), N-vinylpyrrolidone (NVP), and hydroxyethyl methacrylate (HEMA) [19,20]. However, these monomers exhibit low boiling points, leading to significant volatilization during urushiol curing (typically conducted at temperatures ranging from 120 °C for 10 h to 180 °C for 1 h), and their general incompatibility with urushiol may further complicate the copolymerization process. Therefore, identifying an appropriate monomer modifier is a crucial challenge for achieving hydrophilization of urushiol-based coatings via high-temperature copolymerization.

To date, research on the easy-cleaning properties of urushiol-based coatings remains limited, and the introduction of hydrophilic modifiers into urushiol-based materials has primarily relied on physical methods such as blending [21,22,23]. In this study, polyethylene glycol monooleate (PEGMO) was employed as a macromonomer modifier to enhance the hydrophilicity of urushiol-based coatings, resulting in the creation of a novel urushiol-based copolymer coating through thermal curing. PEGMO’s high molecular weight prevents volatilization, making it particularly suitable for copolymerization with urushiol at high temperatures. Furthermore, PEGMO consists of polyethylene glycol (PEG) and oleate ester, where PEG imparts good hydrophilicity due to its strong affinity for water, while the C_18_ unsaturated aliphatic hydrocarbon in oleate ester, similar to urushiol’s side chain, improves compatibility and facilitates copolymerization. The surface hydrophilicity and the oil detachability of the modified coating were systematically investigated by measuring critical parameters such as the contact angles, adhesion work, residual work, and adhesive force. Based on these findings, the coating’s cleaning performance was evaluated using a simple water rinsing method, and its physical and mechanical properties were also examined.

## 2. Materials and Methods

### 2.1. Materials

Urushiol was extracted from oriental lacquer obtained from the Xi’an Institute of Lacquer (Xi’an, China) using the ethanol extraction method, yielding a solid content of 92%. PEGMO, with a molecular weight of 400, along with other analytical grade reagents including xylene (99%), diiodomethane (98%), and hexadecane (98%), were purchased from Aladdin Reagent Co., Ltd. (Shanghai, China) and used as received.

### 2.2. Preparation of Urushiol-Based Coatings

Urushiol and PEGMO were mixed into xylene solvent and dissolved with magnetic stirring to create a 30 wt% coating solution. This solution was uniformly applied to glass slides (for structural and surface property characterization) or tinplate substrates (for physical and mechanical properties testing) using a 100 μm film applicator. Subsequently, the coated substrates were then placed flat in an oven and subjected to thermal curing at 140 °C for 12 h (pure PEGMO volatilizes approximately only 1.5 wt% under these conditions). Following this process, urushiol-based coatings were produced. The mass ratios of urushiol to PEGMO were varied at 100/0, 90/10, 80/20, 70/30, and 60/40, and their prepared coatings were designated as PU, P(U-10PEGMO), P(U-20PEGMO), P(U-30PEGMO), and P(U-40PEGMO), respectively.

### 2.3. Characterization

The chemistry of urushiol-based coatings was analyzed using infrared spectroscopy (IR, iS5, Thermo Nicolet, Madison, WI, USA) and X-ray photoelectron spectroscopy (XPS, Axis Ultra DLD, Kratos Analytical, Manchester, UK). The glass transition temperature (*T*_g_) of the coatings was measured using a dynamic thermomechanical analyzer (DMA, Q800, TA Instruments, New Castle, DE, USA) under conditions of a frequency of 1 Hz and a heating rate of 3 °C/min. The cross-sectional morphology of the coatings was examined using a scanning electron microscope (SEM, SU8010, Hitachi, Hitachinaka, Japan). A thermogravimetric analyzer (TG, TG209F3, Netzsch, Braunschweig, Germany) was utilized to investigate the thermal stability of the coatings under conditions of a nitrogen atmosphere and a heating rate of 10 °C/min.

### 2.4. Surface Wettability Testing and Theoretical Calculations

Contact Angle Measurement: the water contact angle and underwater oil (hexadecane) contact angle were measured using a contact angle goniometer (JC2000D1, Shanghai Zhongchen Digital Technology Equipment Co., Ltd., Shanghai, China). A 2 μL droplet of water or oil was placed on the coating surface, with each contact angle recorded at least five times to obtain an average value. To assess durability under harsh conditions, the coating was immersed in aqueous solutions (pH = 4, pH = 10, and 10% NaCl) for 72 h. Water contact angles were measured before and after immersion to analyze performance.

Surface Energy Calculation: Using deionized water and diiodomethane as test liquids, the contact angles of both on the coating surfaces were measured independently. The surface energy (*γ*) of the coatings, along with its polar (*γ*^p^) and dispersion (*γ*^d^) components, was then calculated using the Owens-Wendt-Rabel-Kaelble (OWRK) method [24,25].

Calculation of Adhesion work (*W*_a_) and Residual work (*A*_R_): Based on the measured underwater oil contact angle (*θ*_ow_), the values of *W*_a_ and *A*_R_ of the oil droplet on the coating surface were calculated according to Equation (1) [26] and Equation (2) [27], respectively:

(1) $$W_{a}=\gamma_{ow}(cos\theta_{ow}+1)$$

(2) $$A_{R}=\pi {\left(\frac{3v}{\pi }\right)}^{2/3}\left(\sqrt[{3}]{4}-\sqrt[{3}]{2-3\cos\theta_{ow}+cos{\theta_{ow}}^{3}}\right)\gamma_{ow}$$

where the interfacial free energy (*γ*_ow_) between hexadecane and water is 53.3 mJ/m^2^ at room temperature (25 °C) [28] and the oil droplet volume (*v*) is 2 μL.

Adhesive Force Measurement: Following the principle outlined in the literature [29,30], the adhesive force was quantified using a high-sensitivity microelectronic balance (Secura125-1CN, Sartorius, Göttingen, NI, Germany). The coating was positioned at the bottom of a transparent container filled with water, placed horizontally on the balance pan. A 5 μL oil droplet of hexadecane fixed to a metal ring was lowered to contact the coating surface, then slowly moved upwards at a speed of 0.05 mm/s until it detached from the surface. The maximum force recorded during detachment was defined as the adhesive force. Shape changes in the oil droplet during the test were captured using a charge-coupled device (CCD) camera system. Each measurement was repeated five times and averaged.

### 2.5. Easy-Cleaning Evaluation

Using hexadecane as a model oil pollutant, the cleaning performance of the urushiol-based coating was evaluated through a simple water rinsing method. A known amount of hexadecane droplets was dripped onto the coating surface. The contaminated coating was then immersed in water and subjected to gentle shaking several times to facilitate oil removal. After the cleaning process, the coating was vacuum-dried at room temperature and weighed [30], and the oil residues (*O*_R_) remaining on the surface were calculated using Equation (3):

(3) $$O_{R}=\frac{W_{2}-W_{1}}{W_{0}}\times 100\%$$

where *W*_0_, *W*_1_, and *W*_2_ represent the weight of the hexadecane droplet, the uncontaminated coating, and the contaminated coating after the cleaning process, respectively. The cleaning experiment was conducted three times, and the results were averaged for accuracy.

### 2.6. Physical and Mechanical Properties Testing

Testing of the coating was conducted according to the following standards: glossiness (GB/T 1743-1989), adhesion (GB/T 9286-1998), flexibility (GB/T 1731-1993), impact resistance (GB/T 1732-1993), and pencil hardness (GB/T 6739-1996).

## 3. Results and Discussion

### 3.1. Characterization of P(U-PEGMO) Coating

**Figure 1:**
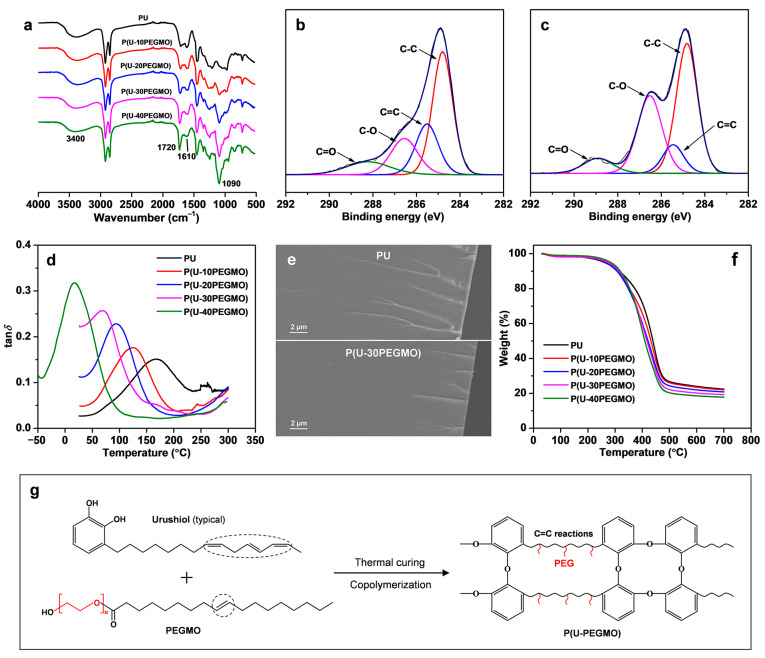
Characterization of urushiol-based coatings: (**a**) IR spectra, (**b**) C1s XPS spectra of PU, (**c**) C1s XPS spectra of P(U-30PEGMO), (**d**) DMA curves, (**e**) SEM images, (**f**) TG curves, and (**g**) proposed reaction scheme between urushiol and PEGMO.

The thermal curing and film-forming process of urushiol is highly intricate, involving oxidative and addition polymerization reactions of its unsaturated side chain [1,4]. When the macromonomer modifier of PEGMO was introduced, copolymerization likely occurred between the side chain of urushiol and the C=C bond of PEGMO during curing, resulting in the formation of a urushiol-based coating. Figure 1a shows the IR spectra of the serial P(U-PEGMO) coatings. The absorption peaks in the spectra and their corresponding functional groups are as follows: 3400 cm^−1^ (O-H stretching vibration), 2925 cm^−1^ (-CH_3_ stretching vibration), 2850 cm^−1^ (-CH_2_ stretching vibration), 1720 cm^−1^ (C=O stretching vibration), 1610 cm^−1^ (benzene ring stretching vibration), 1455 cm^−1^ (-CH_2_ bending vibration), 1350 cm^−1^ (-CH_3_ bending vibration), 1250 cm^−1^ (C-O stretching vibration), 1090 cm^−1^ (C-O-C stretching vibration), 950 cm^−1^ and 850 cm^−1^ (-CH bending vibration), and 725 cm^−1^ (-CH_2_ rocking vibration) [31]. The presence of the urushiol unit in the coatings was confirmed by the characteristic peaks of O-H and benzene ring at 3400 cm^−1^ and 1610 cm^−1^, respectively [16], while the presence of the PEGMO unit was confirmed by the characteristic C-O-C peak at 1090 cm^−1^ [32]. Both units were additionally identified by the characteristic peak of C=O observed at 1720 cm^−1^ [16,32]. Importantly, as the PEGMO content increased, the strength of the 1720 cm⁻¹ and 1090 cm⁻¹ peaks in the coatings intensified, indicating an increasing proportion of the PEGMO unit in the composition.

The chemistry of urushiol-based coatings was further analyzed using XPS, with the results presented in Figure 1b,c (other samples not listed) and summarized in Table 1. The C1s spectrum for the PU coating was deconvoluted into four absorption peaks corresponding to C-C (284.8 eV), C=C (285.5 eV), C-O (286.5 eV), and C=O (288.2 eV) bonds. Among these, the C=C bond primarily originated from the benzene ring retained after the curing of urushiol, while the C-O bond likely resulted from the dehydration condensation reaction of phenolic hydroxyl groups and the oxidative polymerization of side chains during curing [33]. Upon the incorporation of PEGMO, the modified coatings retained these four carbon bond absorption peaks; however, the intensity of their C=C bond absorption peaks decreased. With an increase in PEGMO content, the relative contents of C=C bonds in the coatings dropped sharply (Table 1). This reduction can be attributed to both the reduced proportion of urushiol in the coatings and the introduction of PEGMO, which facilitated the polymerization of urushiol’s unsaturated side chain. Additionally, the intensity of the C-O bond absorption peaks in the coatings increased significantly, undoubtedly due to the incorporation of the PEG segment via the PEGMO modifier. When the modifier content reached 40%, the relative content of the C-O bond in the P(U-PEGMO) coating increased doubled (Table 1).

Figure 1d displays the DMA curves for the PU and P(U-PEGMO) coatings. DMA can assess the glass transition of polymers, thus providing insights into their structure and compatibility [34]. In the figure, the temperature at which the peak of the loss factor (tan*δ*) occurred corresponds to the *T*_g_ of the coating. It is noteworthy that all series of P(U-PEGMO) coatings exhibited a single internal friction peak, signifying the presence of only one *T*_g_. This observation suggests that the modified coating was composed of a single polymeric structure, i.e., the copolymer formed from the reaction between urushiol and PEGMO. The single *T*_g_ also reflects excellent compatibility between the two monomers post-copolymerization, likely due to the structural similarity between the unsaturated side chain (C_15_ or C_17_) in urushiol and the unsaturated aliphatic hydrocarbon segment (C_18_) in PEGMO. Furthermore, an increase in PEGMO content was associated with a gradual decrease in the *T*_g_ of the modified coatings, ranging from 168.9 to 17.4 °C. The higher *T*_g_ of the PU coating can be attributed to its high content of rigid benzene rings, whereas the incorporation of flexible PEG segments in the P(U-PEGMO) coating led to a lower *T*_g_. Further investigation of the compatibility of the coatings was conducted using SEM, as shown in Figure 1e. Similar to the PU coating, the cross-section of the P(U-PEGMO) coating exhibited no delamination or microscopic phase separation, except for fracture lines at the edge resulting from the cross-sectional preparation. The cross-section appeared uniformly dense and compact, reiterating the good compatibility within the modified copolymer coating. Figure 1f demonstrates the TG results for the urushiol-based coatings. All coatings presented weight loss within the temperature range of 200–650 °C. Compared to the PU coating, the P(U-PEGMO) coatings showed a slightly faster rate of weight loss, but their 5% weight loss temperatures were all above 260 °C. This finding indicates that the modified coatings are still suitable for use under higher ambient temperatures and possess good thermal resistance. Based on the above analyses, as illustrated in Figure 1g, it can be inferred that during the thermal curing process, urushiol and PEGMO underwent C=C bond reactions (either oxidative or addition polymerization), leading to a cross-linked urushiol-based copolymer coating with a stable chemical structure and good compatibility. Moreover, the integration of functional PEG segments into the copolymer structure is anticipated to enhance its unique properties.

### 3.2. High Surface Hydrophilicity

**Figure 2:**
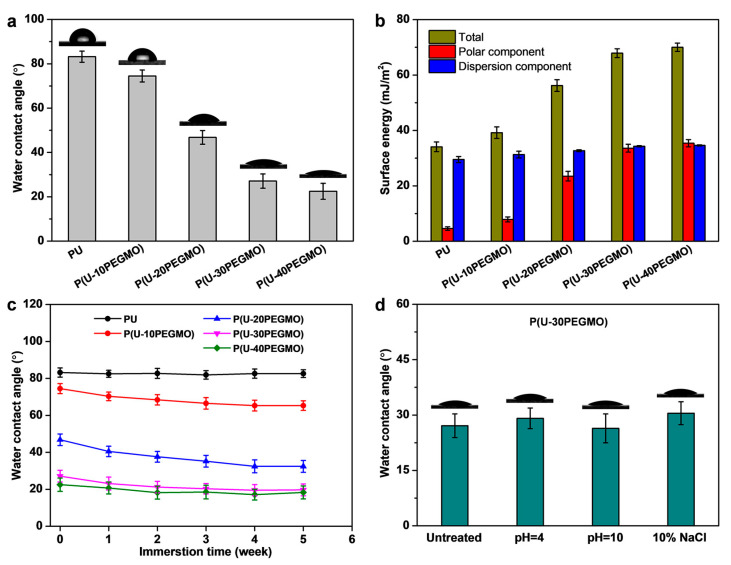
Surface hydrophilicity of urushiol-based coatings: (**a**) water contact angles, (**b**) surface energy, and changes in contact angles after (**c**) prolonged water immersion and (**d**) exposure to harsh conditions.

Contact angle measurement is an effective quantitative method for evaluating the surface wettability of polymer materials. Figure 2a shows the water contact angles of the urushiol-based coatings. The contact angles progressively decreased from 83.2° for PU to 74.5°, 46.8°, 27.1°, and 22.5° for P(U-10PEGMO), P(U-20PEGMO), P(U-30PEGMO), and P(U-40PEGMO), respectively. The PU coating exhibited limited surface hydrophilicity due to its structural backbone, which primarily consists of non-polar benzene rings and aliphatic hydrocarbons, with only a small amount of polar oxygen-containing groups [35]. In contrast, the introduction of PEGMO macromonomer greatly enhanced the surface hydrophilicity of the modified coatings, correlating with an increased PEGMO content. This enhancement stems from the incorporation of the strongly polar PEG segment in the macromonomer (Figure 1g). When PEG interacts with water, its oxygen atoms readily form hydrogen bonds with the water molecules, allowing the PEG chain to tightly bind with water and demonstrating superior hydrophilicity [36]. Furthermore, research has demonstrated that surface hydrophilicity is highly dependent on the density of polar groups present on the material’s surface [37]. Consequently, as the PEGMO content reached 30% or higher, the contact angle of the modified coating dropped sharply to below 30°, indicating a high degree of hydrophilicity. This remarkable improvement can be attributed to the significantly increased content of surface polar C-O-C groups (Figure 1c and Table 1).

To further evaluate surface wettability, the surface energy of the urushiol-based coatings was calculated, with results shown in Figure 2b. The surface energy of the P(U-PEGMO) coatings increased dramatically compared to that of the PU coating. This increase is primarily ascribed to the enhanced polar component of the surface energy, which reflects interactions between polar molecules such as hydrogen bonding, dipole–dipole forces, and induced forces [25]. With the increase in PEGMO content, the polar component of the modified coatings’ surface energy rose steadily from 4.6 mJ/m^2^ to over 30 mJ/m^2^, while the dispersion component remained relatively constant. These findings confirm that the incorporation of PEG segments enhanced the molecular polarity of the coatings, thereby improving their surface hydrophilicity. The observed trend in surface energy changes shown in Figure 2b closely corresponds with the contact angle variations depicted in Figure 2a.

The durability of the coatings’ surface hydrophilicity was investigated by monitoring changes in contact angles after prolonged water immersion and exposure to pH = 4, pH = 10, and 10% NaCl aqueous solutions. As presented in Figure 2c, the contact angles of the PU coating remained largely unchanged over time. Interestingly, the contact angles of the serial P(U-PEGMO) coatings gradually decreased with prolonged immersion, indicating a slight, further improvement in the surface hydrophilicity. Furthermore, as shown in Figure 2d, the contact angle variation of the P(U-30PEGMO) coating was minimal, maintaining a value below 31° under the harsh conditions of acid, alkali, and saline exposure. These findings demonstrate the excellent durability of the modified coatings’ surface hydrophilicity across diverse environmental conditions. This durability can be attributed to the stable, crosslinked chemical structure formed during the thermal curing process, where PEGMO covalently bonds with the urushiol matrix (Figure 1g). This robust structure effectively prevents the detachment of modifiers, ensuring long-lasting hydrophilicity. Additionally, it is speculated that during prolonged immersion, the PEG segments within the modified coating migrated towards the surface in response to water orientation, leading to the enhanced surface hydrophilicity of the coatings [38].

### 3.3. Oil Detachability

The detachability of oil pollutants from the surfaces of a coating is a key factor in its easy-cleaning performance. Using hexadecane as a model oil, the oil detachability of the P(U-PEGMO) coating was systematically studied through the oil contact angle, adhesion work, residual work, and adhesive force.

**Figure 3:**
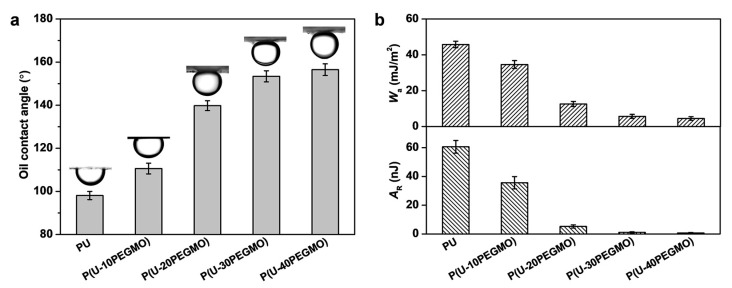
(**a**) Underwater oil contact angles and (**b**) adhesion work and residual work of urushiol-based coatings.

The underwater oil contact angle serves as a useful metric to evaluate the oleophobicity of a material’s surface in an aqueous environment. Stronger oleophobicity suggests that oil pollutants are more easily removed during water-based cleaning processes [15]. As shown in Figure 3a, the PU coating displayed a relatively low underwater oil contact angle of 98.1°, indicating a weak oleophobic characteristic. In contrast, as the PEGMO content increased, the P(U-PEGMO) coatings exhibited a progressive increase in oil contact angles. At the modifier content of 30% or higher, the oil contact angle of the coating exceeded 150°, demonstrating underwater superoleophobicity and indicating that oil droplets could be easily detached from the surface. This enhanced underwater oleophobicity is primarily attributed to the hydrophilic nature of the coatings’ surface. A hydrophilic surface generates a hydration layer in water, which diminishes the contact area between oil droplets and the solid surface, thus increasing the oil contact angle [39,40]. The relationship between surface hydrophilicity and the underwater oil contact angle can be further elucidated through the application of Young’s equation (Equation (4)) [41]:

(4) $$cos\theta_{ow}=\frac{\gamma_{o}cos\theta_{o}-\gamma_{w}cos\theta_{w}}{\gamma_{ow}}$$

where *θ*_ow_, *θ*_o_, and *θ*_w_ represent the underwater oil contact angle, oil contact angle in air, and water contact angle in air on a solid surface, respectively. Meanwhile, *γ*_o_*, γ*_w_, and *γ*_ow_ stand for the surface tension of the oil, the surface tension of the water, and oil–water interfacial tension, respectively. Since the value of *γ*_w_ (72.0 mN/m at room temperature) is significantly greater than that of *γ*_o_ (27.5 mN/m at room temperature for hexadecane), it can be deduced for a hydrophilic surface (where *θ*_w_ < 90°) that *γ*_o_ cos*θ*_o_ − *γ*_w_ cos*θ*_w_ < 0, leading to *θ*_ow_ > 90°. This indicates that a hydrophilic surface exhibits oleophobicity underwater, and as the hydrophilicity increases (i.e., *θ*_w_ decreases), the underwater oleophobicity improves (i.e., *θ*_ow_ increases). Based on this theoretical framework, the P(U-PEGMO) coatings with highly hydrophilic surfaces (where *θ*_w_ < 30°, as shown in Figure 2a) could demonstrate superoleophobicity underwater.

Using the aforementioned underwater oil contact angle values, the adhesion work and residual work of oil droplets on the coating surfaces can be calculated, with the results shown in Figure 3b. Adhesion work refers to the work required by an external force to separate a solid–liquid interface of a unit area from the junction [42]. Residual work, a concept in the principle of washing and decontamination, is the external work needed to drive the contact angle of an oil droplet from its equilibrium value to 180°, allowing the oil droplet to completely detach from the solid surface through the “rolling up” mechanism [27,43]. Therefore, both the adhesion and residual work can be used to assess the oil detachability from a thermodynamic perspective. As seen in Figure 3b, the P(U-PEGMO) coatings exhibited greatly lower adhesion and residual work compared to the PU coating, with their values decreasing to below 6.0 mJ/m^2^ and 1.5 nJ, respectively, when the PEGMO content reached 30% or higher. This finding suggests that oil droplets can detach from the coating surface with minimal external energy input.

**Figure 4:**
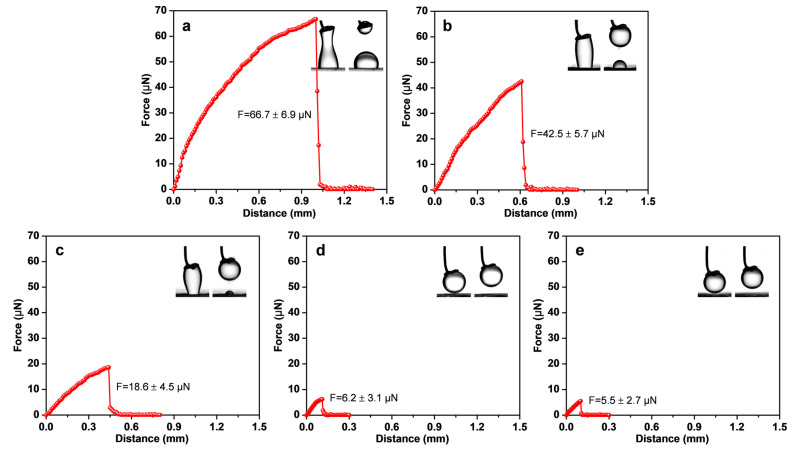
Force–distance curves and images during adhesive force tests when oil droplets detached from urushiol-based coating surfaces: (**a**) PU, (**b**) P(U-10PEGMO), (**c**) P(U-20PEGMO), (**d**) P(U-30PEGMO), (**e**) P(U-40PEGMO).

Furthermore, the oil adhesive forces on the coating surfaces were measured using a microelectronic balance system. The adhesive force refers to the actual force required to detach a specific oil droplet from a solid surface, enabling a quantitative assessment of oil detachability. Figure 4 presents the oil adhesive force test curves and images of the oil droplet detachment process. The maximum force recorded in the curve represents the oil adhesive force. Notably, the PU coating showed a high adhesive force of approximately 70 μN, with the oil droplet becoming severely distorted and elongated, leaving a significant residue on the surface. This observation indicates a strong adhesion of the oil droplets to the PU surface. In contrast, as the PEGMO content increased, the P(U-PEGMO) coatings displayed a gradual decrease in adhesive forces, with less distortion of the oil droplets and reduced residual oil. Ultimately, the adhesive forces for P(U-30PEMMO) and P(U-40PEGMO) coatings dropped to 5–6 μN, and the oil droplets maintained their spherical shape immediately upon detachment without any residue. This suggests that the oil droplets were less prone to adhering to the modified coating surfaces. The hydrated layer formed on these coating surfaces acts as a barrier, effectively inhibiting the adhesion of oil droplets [44]. In summary, the analyses of the underwater oil contact angle, adhesion and residual work, and adhesive force confirm that high hydrophilicity (with a contact angle below 30°) significantly facilitates the ease of oil detachment from the modified coating surface.

### 3.4. Easy-Cleaning Performance

**Figure 5:**
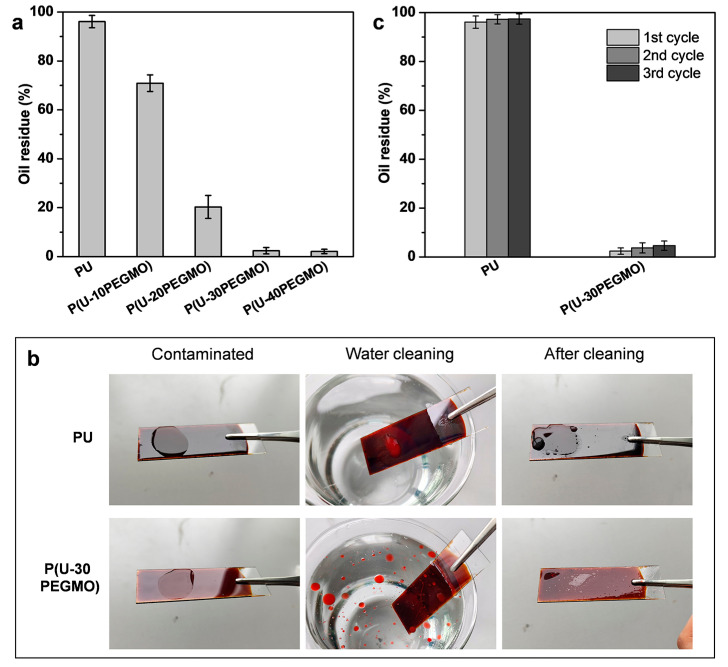
Cleaning performance of urushiol-based coatings: (**a**) oil residues after cleaning, (**b**) images of the cleaning process (glass slide dimensions: 25 mm × 75 mm), and (**c**) oil residues after repeated fouling and cleaning cycles.

Building on prior studies of the surface wettability and oil detachability, the cleaning performance of the P(U-PEGMO) coating was investigated using a simple water rinsing method. In the experiment, a measured amount of hexadecane was applied as a model oil to contaminate the coating. After water rinsing, the residual oil remaining on the coating surface was weighed to quantitatively evaluate the cleaning efficiency. As shown in Figure 5a, the oil residues on the PU and P(U-10PEGMO) coatings exceeded 70 wt% following the cleaning process, indicating that these coatings were difficult to clean with water alone once contaminated. In contrast, when the PEGMO content was increased to 30% or higher, the P(U-30PEGMO) and P(U-40PEGMO) coatings demonstrated significantly reduced oil residues of only 2.4 wt% and 2.1 wt%, respectively, highlighting their excellent easy-cleaning properties. Figure 5b shows images of the cleaning process on the coating surfaces (with oil droplets dyed red for clarity), allowing for further insight into their cleaning behaviors. After a simple water rinse, oil droplets remained tightly adhered to the PU surface without detachment. Conversely, when the P(U-30PEGMO) coating was contaminated with oil and subjected to gentle shaking in water, a substantial amount of oil pollutants detached from its surface and floated in the water. Following cleaning, a layer of water film formed on the coating surface, leaving no visible oil residue. The superior easy-cleaning performance of the modified coatings can be attributed to their high surface hydrophilicity (Figure 2a). When contaminated with oil and rinsed with water, the highly hydrophilic coating surfaces readily hydrate to form stable and dense hydration layers, which impart strong oil-repellent properties (Figure 3a) and greatly reduce oil droplet adhesion (Figure 4). As a result, only a minimal external force, such as gentle shaking in water, is required to replace the coating/oil interface with the coating/water interface, thereby achieving effective surface cleanliness.

To assess the durability of the easy-cleaning properties of the P(U-PEGMO) coating, repeated fouling and cleaning experiments were conducted over three cycles. The oil residues on the coating surfaces after each cleaning cycle are shown in Figure 5c. The PU coating retained over 95 wt% of oil pollutants after each cleaning, further confirming the absence of its easy-cleaning properties. In contrast, the oil residues on the P(U-30PEGMO) coating consistently remained below 5 wt% throughout the three cycles, demonstrating stable and long-lasting easy-cleaning properties. This durability is likely due to the robust bonding between PEGMO and the coating, which resists being washed away and helps maintain the coating’s surface hydrophilicity.

### 3.5. Physical and Mechanical Properties

In addition to its surface easy-cleaning capabilities, the physical and mechanical properties of the P(U-PEGMO) coating are crucial for its practical applications. Table 2 summarizes the results of basic physical and mechanical tests conducted on the coatings. Compared to the PU coating, the P(U-PEGMO) series maintained similarly high glossiness, indicating that the modified coatings preserved a smooth and flat surface. Moreover, the adhesion, flexibility, and impact resistance of the modified coatings improved greatly, though their hardness decreased. Notably, the pencil hardness of the P(U-40PEGMO) coating dropped to grade HB, which, despite its excellent easy-cleaning properties (Figure 5a), limits its suitability for certain practical contexts. These changes in physical and mechanical characteristics are primarily attributed to the incorporation of PEG segments into the coating structure [45]. The strong polarity of PEG enhanced the adhesion of the coating, while its flexible chain improved the coating’s flexibility and impact resistance, albeit at the cost of hardness. The observed reduction in hardness may also be associated with a decreased crosslinking density within the urushiol film following copolymerization. Overall, the P(U-30PEMMO) coating demonstrated a well-balanced performance, with adhesion, flexibility, impact resistance, and hardness values of grade 2, 2 mm, 90 cm, and grade 2H, respectively. In addition to these favorable mechanical properties, it also exhibited high surface hydrophilicity and excellent easy-cleaning performance (Figure 2a and Figure 5).

## 4. Conclusions

In this study, a urushiol-based coating, P(U-PEGMO), was prepared through thermal curing using the hydrophilic macromonomer PEGMO as a modifier. During the curing process, urushiol and PEGMO reacted to form a copolymer structure with excellent compatibility, facilitated by the interaction between urushiol’s unsaturated side chain of and PEGMO’s C=C bond. At a PEGMO content of 30% or higher, the water contact angle on the surface of the modified coating was less than 30°, signifying a high degree of hydrophilicity due to the incorporation of numerous PEG segments into the coating matrix. Furthermore, the hydrophilicity of the modified coating demonstrated good durability. Benefiting from the high hydrophilicity, the modified coating exhibited an underwater oil contact angle exceeding 150°, an oil adhesive force as low as 5–6 μN, and reduced oil adhesion work and oil residual work, thereby indicating robust underwater oil-repellent properties. After a simple rinsing with water, the oil residues on the P(U-30PEMMO) and P(U-40PEGMO) coatings were only 2.4 and 2.1 wt%, respectively, whereas the PU coating showed no comparable cleaning performance. It can be inferred that the easy-cleaning properties of the modified coating can be attributed to its high surface hydrophilicity. Moreover, in comparison to the PU coating, the P(U-PEGMO) coatings showed improved flexibility, impact resistance, and adhesion, albeit with slightly reduced hardness. This study proposes a convenient and effective strategy for addressing oil contamination in natural coatings, thereby broadening their potential application scenarios.

## Figures and Tables

**Table 1:** Relative content of different forms of carbon in urushiol-based coatings obtained from the XPS results.

Coating	Carbon Atomic Content (%)			
	C-C	C=C	C-O	C=O
PU	47.9	22.2	19.0	10.9
P(U-10PEGMO)	47.6	20.2	19.1	13.1
P(U-20PEGMO)	49.5	16.5	24.8	9.2
P(U-30PEGMO)	48.6	10.5	33.1	7.8
P(U-40PEGMO)	46.8	9.0	38.2	6.0

**Table 2:** Physical and mechanical properties of urushiol-based coatings.

Coating	Glossiness/%	Adhesion	Flexibility/mm	Impact Resistance/cm	Pencil Hardness
PU	110.5 ± 2.5	4	10	6	5H
P(U-10PEGMO)	109.9 ± 2.8	3	5	9	3H
P(U-20PEGMO)	107.5 ± 3.2	3	3	85	3H
P(U-30PEGMO)	106.1 ± 3.7	2	2	90	2H
P(U-40PEGMO)	102.9 ± 3.8	2	1	88	HB

## Data Availability

The data underlying this study are available in the published article; further inquiries can be directed to the corresponding authors.
